# Training for Tuberculosis Elimination in Indonesia: Achievements, Reflections, and Potential for Impact

**DOI:** 10.3390/tropicalmed4030107

**Published:** 2019-07-18

**Authors:** Stephanie Main, Trisasi Lestari, Rina Triasih, Geoff Chan, Lisa Davidson, Suman Majumdar, Devy Santoso, Sieyin Phung, Janne Laukkala, Steve Graham, Philipp du Cros, Anna Ralph

**Affiliations:** 1 International Development, Burnet Institute, Melbourne VIC 3000, Australia; 2 Menzies School of Health Research, Darwin 0811, Australia; 3 Centre for Tropical Medicine, Universitas Gadjah Mada, Yogyakarta 55281, Indonesia; 4 Department of Pediatric, Dr. Sardjito Hospital/Faculty of Medicine, Public Health and Nursing, Universitas Gadjah Mada, Yogyakarta 55281, Indonesia; 5 Australia Awards in Indonesia, Jakarta 12940, Indonesia; 6 Centre for International Child Health, The University of Melbourne and Murdoch Chldren’s Research Institute, Parkville VIC 3050, Australia

**Keywords:** tuberculosis, elimination, health workforce, capacity building, training, impact

## Abstract

Indonesia has the third highest tuberculosis (TB) caseload internationally. A cornerstone for strengthening health systems to respond to TB is a well-trained workforce. In a partnership between Indonesian and Australian institutions, TB training was run during 2018 to strengthen the local capacity to meet End TB strategy targets. This paper aims to report on course design, delivery, training outcomes, and reflections. Seventy-six Indonesian healthcare workers, program staff, researchers, and policy-makers were selected from over 800 applicants. The structure comprised three trainings, each with a pre-course workshop (in Indonesia) to identify learning needs, a two-week block (Australia), and a post-course workshop (Indonesia). The training content delivered was a combination of TB technical knowledge and program/project theory, design, and logic, and the training utilised multiple teaching and learning methods. An innovative element of the training was participant-designed TB workplace projects focusing on context-specific priorities. Evaluation was undertaken using participant surveys and appraisal of the projects. Participants rated the course highly, while success in project implementation varied. Reflections include the importance of involving Indonesian experts in delivery of training, the need to understand participant learning requirements and adapt the training content accordingly, and the challenge of measuring tangible training outputs.

## 1. Introduction

The global End Tuberculosis (TB) strategy sets ambitious goals for achieving TB elimination by 2035. Moving from TB control to TB elimination requires shifts in policy, implementation, and capacity, along with new strategies, models of care, and increased resources [1]. Human resource constraints are a key barrier to TB elimination. There have been calls for an urgent increase in the quality, quantity, distribution, and management of healthcare workers to meet the End TB targets [2,3]. Inadequate investment in training have been recognized as key barriers to TB elimination [4]. Healthcare workers need to be equipped to adapt to new evidence and programmatic changes [5].

Approximately 8% of the total 10 million TB cases occurred in Indonesia in 2017, which has the third-highest global TB caseload, and is recognised by the World Health Organization (WHO) as a high-burden country for TB, Multi-Drug Resistant TB (MDR-TB), and TB/HIV [6]. Human resource development, such as in-service training, training for remote-area staff, management skills, and capacity for HIV and MDR-TB care have been noted as sub-optimal and remain a challenge for TB elimination in Indonesia [7]. In 2018, in response to these ongoing challenges, three cohorts of Indonesian health workers, program managers, researchers, and policy makers attended training courses that focussed on “Tuberculosis: Prevention and Elimination”. The courses were delivered by Australian institutions (Menzies School of Health Research and Burnet Institute) in partnership with Universitas Gadjah Mada (UGM), Indonesia.

This commentary reports on the three courses delivered under this program. The paper aims to provide a description of the purpose, design, and delivery of the training; reflect on lessons learned; discuss the effectiveness of training methodologies; and highlight key challenges in evaluating trainings.

## 2. Indonesian TB Elimination Training

### 2.1. Training Overview

The TB elimination training was a series of three Australia Awards in Indonesia (AAI) short courses funded by the Australian Government. AAI is a long-running program that coordinates scholarships and courses for Indonesian participants across a range of subjects to equip individuals with knowledge and skills to contribute to positive change in their work [8]. TB training had not previously been offered by AAI, but was recognized as an area of high need by partner organizations and the Australian Government.

The structure and key content areas were determined by the Australian Government department funding the course. The program’s logic, including the course goal and objectives, were pre-selected by AAI and identified through consultations with the Indonesian Ministry of Health and the Australian embassy in Jakarta. The interpretation of specific topics and delivery design was then developed by the authors of this paper, and revised during the running of the three sequential courses in response to feedback.

Problems in Indonesia’s response to TB included under-resourcing relative to the disease burden, and lack of highly skilled experts across the very geographically dispersed nation [7]. In particular, eastern Indonesia (Papua province) is recognized as being an especially high-burden region, with low socioeconomic status and geographical and language barriers to health care. Specific deficits in TB control are inadequate case-finding, and poor participation in recommended diagnostic, notification, and management guidelines by private practitioners [7,9].

In response, AAI training prioritized having a focus on preventive therapy implementation, active case-finding, case reporting, and public–private engagement. Participants from minority ethnicities within Indonesia, especially from eastern provinces, were prioritized for selection.

Table 1 summarizes major components of the training. Proceeding sub-sections describe characteristics of the participants selected, and an evaluation of the training based on course outcomes.

### 2.2. Participant Characteristics

Of 817 applications, 76 Indonesian doctors, nurses, other health workers, program managers, researchers, and policy-makers were selected for the trainings (i.e., 25 or 26 per course) based on the above-described criteria. The median (IQR) age of participants was 40 (34–48) years, and 45 (59%) were female. The majority (45/76, 59%) held a Master’s degree in a health-related field as their highest qualification. Participants were from governmental, private, and non-government sectors. The governmental sector was the predominant sector which was represented (36 [47%] participants), a third of whom (13/36) worked for the Ministry of Health. Most common provinces of origin were the most populous province, Java (41 [54%]) and the highest TB-burden province, Papua (20 [26%]).

### 2.3. Training Evaluation: Acceptibility, Relevance, Learning, and Links Forged

Seventy-five participants completed pre- and post-training learning evaluations. The median self-perceived knowledge increase was 3 out of 10 points on all 15 topics of the training (Table S1). On average, participants strongly agreed that the training was acceptable and relevant to their work, and that they were satisfied with the content and training overall (Table S2).

Development of professional links could not be objectively measured, but this outcome was seen to be achieved by the highly-rated site visits (from participant surveys), the networking events organized, and the communication platforms, such as the “WhatsApp^TM^” network and ongoing quarterly course newsletter.

### 2.4. Training Evaluation: TB Elimination Projects

Projects ranged from health promotion tools to active case-finding intiatives using phone applications. Table S3 summarises the 18 projects which were developed and implemented by thematic areas. One group which developed a treatment adherence tool won an award from the Indonesian TB Research Network and received further support through the AAI Alumni Grant Scheme for a scale-up of the project [14].

## 3. Strengths and Limitations of the Trainings

### 3.1. Involving Local Experts in Country Specific Trainings

To ensure locally specific, relevant content, the training team included Indonesian TB experts in all parts of the three courses, and invited speakers from the Indonesian National TB Program. These experts provided in-depth knowledge of the TB and health systems in Indonesia and associated challenges with the program, implementation, and research. This was fundamental to participants engaging with the content, understanding, and participating in their learning. Involvement of local experts also strengthened partnerships between Australian and Indonesian institutions.

### 3.2. Participant Selection

The participant selection process aimed to ensure that those selected were likely to make a positive difference to local TB programs and outcomes to maximize the impact of the training [8]. Diversity in the participants’ backgrounds enriched the training by providing exposure in classroom discussions to different perspectives and experiences. However, the diversity in baseline knowledge and learning requirements also posed challenges, but individual learning needs were able to be addressed, particularly during break-out group work.

### 3.3. Training Methodology

A combination of technical content and program theory/logic, delivered using participatory learning, problem-solving, and critical thinking were fundamental elements of the training and contributed to positive feedback. The three-phase design allowed reworking of content to adapt to key identified needs. These approaches were acknowledged by some participants with teaching roles as approaches they would utilize in their own classrooms in Indonesia.

Australian and Indonesian stakeholders agreed that short courses would be the most suitable modality to respond to the participants’ and their organizations’ learning objectives, for two reasons: firstly, short courses support mid-career professionals who would not be able to leave their work for longer than two weeks to attend a specialized training program; and secondly, short courses provide participants with opportunities to connect with TB specialists from Australia and Indonesia, compare programs and initiatives, and discuss government policies and frameworks. Based on studies of short AAI courses carried out by AAI with multi-sector participants, one of the long-term outcomes of the courses is the trust built between the participants, enabling them to share ideas openly and express their opinions about government policies.

A key limitation identified was that this was predominantly classroom-based learning, rather than on-the-job learning.

### 3.4. Training Evaluation and Measuring Impact

Evaluation measures comprised surveys and outcomes of project implementation able to be assessed at the post-course workshops (Table 1). The projects were intended as a practical mechanism to support change of practices in workplaces, aiming to improve the quality of TB control and service delivery. The degree to which projects were successfully implemented (Table S3) therefore provided a measure of the practical impact of the training. Project implementation varied among the 18 executed projects, from still being in a start-up phase by the end of the training period, to being launched with data collection underway.

Training surveys were administered to ask participants to evaluate acceptability, relevance, and sustainability of the training and learning. The pre- and post-training surveys provided an important indication of satisfaction and knowledge gain. However, they were unable to assess the genuine impact of the training on change, if any, in workplace practices. AAI conducts annual follow-up alumni surveys and sector studies based on requests from the Australian Government. The Kirkpatrick framework is utilized in these surveys, although these primarily focus on the reaction and learning levels [15]. Some work is needed to ensure that assessment of the training and course goals are done using the higher levels of the framework.

## 4. Discussion

The AAI TB elimination courses illustrate challenges and opportunities in seeking to provide effective strengthening of the TB workforce through training. The AAI short courses were well-rated by participants, generated professional Indonesian–Australian connections, and supported the implementation of context-specific projects towards TB elimination. However, the design of the course (being delivered from central locations rather than in the workplace), the scope of the evaluation (being limited to feedback from participants and trainers themselves), and timing of the evaluation (at the final post-course workshop) meant that impact on TB program outcomes could not be measured.

Globally, a considerable amount of foreign aid is invested in health workforce training [16,17], with organisations such as the KNCV (Koninklijke Nederlandse Centrale Vereniging) Tuberculosis Foundation and The International Union Against Tuberculosis and Lung Disease having extensive experience in priovision of TB training. While trainings which have been designed and delivered well have the potential to be extremely valuable for achieving TB elimination, ineffective methodologies and irrelevant content can be problematic and waste vital time and resources [16].

Strengths of the AAI ‘Tuberculosis: Prevention and Elimination’ training courses included appropriate participant selection, effective participatory training methods, and delivery of appropriately-pitched, evidence-based TB course material based on key learning needs. Processes that emphasise skill development in problem-solving and prioritise “learning by doing” are useful in increasing participant capacity, learning, and behaviour change [16,18]. This concept underpins the inclusion of project development and implementation in AAI courses. Table 2 provides a valuable starting point for the development and delivery of future trainings, summarizing key components utilising both pedagogical theory and experience from this training. 

A limitation of this commentary paper is the lack of downstream, longer-term impact data, which was beyond the scope of this training to evaluate. We were able to measure proximal outcomes, including participant satisfaction and knowledge gain, and the extent to which project implementation had occurred by the time of the post-course workshop (2–3 months later) based on self-reported outcomes. In the design of the training, it was hoped that inclusion of award projects would serve as a more useful evaluation for the course by tracking implementation and outcomes from the projects. However, evaluating the implementation of projects without resources, particularly funding and staff, over a short time was challenging. Therefore, the actual change in practice in the delivery of TB control activities as measurable through performance indicators, such as TB notifications or outcomes, cannot be reported in this paper.

Such challenges are in keeping with other findings. Evaluations of HIV, TB, and malaria most commonly focus on pre/post-tests, focusing on factual knowledge [19]. A systematic review indicated that of the large number of TB health worker trainings that occur annually, only a small number have actually been evaluated [20]. Of those evaluated trainings, only three were conducted in the Asia-Pacific region, and these only assessed perceived learning, not behaviour change, performance, or programmatic impact [20].

An approach to testing healthcare provider behaviour change is with the use of standardised patients (actors) to assess diagnosis and management practices after receipt of training [21,22]. A pilot study in India utilised standardised patients to assess TB care across health facilities, demonstrating effectiveness of the methodology [23]. However, this approach is limited to assessing behaviour changes in health workers only.

The Structured Operational Research and Training IniTiative (SORT IT) provides an example of a training model which is inherently structured to evaluate outcomes [24,25]. This model uses on-the-job-training (a “learning by doing” methodology) [26,27,28,29], and supports participants to conduct an ethics-approved project and produce a peer-reviewed publication. While the training offered under the AAI scheme used a different model, some parallels exist, including the participants’ outputs as opportunities to translate learning into practical action.

## 5. Conclusions

The training run under the auspices of the Australia Awards in Indonesia resulted in the implementation of 18 projects addressing key Indonesian and regional priorities in TB elimination. This model used a classroom-based approach, but provided an innovative mechanism to translate learning into practice by utilising participatory learning and supporting the development and implementation of a project in participants’ workplaces. The model offers one approach to human resource capacity-building for TB programs, but would be further strengthened through additional on-the-job training, more robust evaluation frameworks, and needs to occur within a program of innovation and multi-sectoral health system strengthening.

## Figures and Tables

**Table 1:** Overview of training methodology.

Training Component	Description
**Training Goal, Objectives, Outcomes** (pre-specified by AAI)	Goal: The capacity of health workers, program managers, researchers, and policy-makers in Indonesia to end the tuberculosis (TB) epidemic is strengthened through partnership with Australian professionals and institutions. Objectives: The alumni use acquired knowledge, attitudes, and skills to influence their professional fields and communities; The developed projects are used to inform TB policy and practice in Indonesia and positively impact the local response to TB elimination; Alumni draw on developed links and networks to source required support and expertise. Outcomes: Participants gain technical and programmatic knowledge and skills in the latest, evidence-based: ○ Public health principles for TB elimination ○ Clinical care for patients with TB, MDR-TB, TB/HIV, and other co-morbidities ○ TB prevention and management ○ Health-system strengthening and public–private engagement ○ Health promotion and effective Information, Education and Communication (IEC) tools for TB Course participants design, develop, and implement a project in their workplaces based on their learning and problem analyses Professional people-to-people and institutional links are developed between course alumni and facilitators/institutions
**Delivery**	Each of the short courses were split into three parts: a three-day pre-course workshop in Indonesia; a two-week course in Australia several weeks later; and a three-day post-course workshop in Indonesia 2–3 months later [8]. The purpose of this time-frame was to provide participants with opportunities to implement their projects, and reduce disruption to their workplace commitments. Language translation between Indonesian and English was provided simultaneously using on-site translators with headphones for participants and presenters, and course materials were translated and provided to the participants electronically.
**Course Team**	Two course leaders and two designers (medical doctors with TB expertise), one course coordinator (project manager), and a welfare officer from Australian institutions, plus two Australian and two Indonesian course facilitators made up the course team. The core team oversaw and coordinated the design, delivery, and evaluation of the three short courses. The multidisciplinary course delivery team included nurses, epidemiologists, public health physicians, laboratory scientists, advocacy experts, program managers, and health promoters, delivering sessions that covered different areas of expertise.
**Design**	The courses were designed by AAI in response to specific learning objectives identified by the Indonesian Ministry of Health and the Australian Embassy in Jakarta. The training used best-practice teaching and learning methods, including problem-based learning, facilitated interactive discussions, group sessions for problem analysis and solution design, and presentations on key concepts. Lectures, debates, and expert question-and-answer sessions were used to introduce course content. Group participatory sessions were intended to encourage peer learning and help participants apply information to their own contexts. Practice in analysing regionally-relevant scenarios and applying potential solutions was included throughout. Each day commenced with a review of key principles and learning from the previous day. Site visits to TB control programs in Australia (Victoria and Northern Territory), reference laboratories, research institutions, and tertiary hospitals were also included to demonstrate practical applications of interventions and strategies for TB. Networking events were held to develop linkages between participants and experts in Indonesia and Australia. An online participant network platform was formed for ongoing communication.
**Content**	The training content was a combination of technical knowledge and program/project theory, design, and logic. Training comprised a comprehensive technical overview of TB in Indonesia and the region, focusing on key evidence and strategies for TB elimination. Content included drug-resistant TB care, preventive therapy, paediatric TB diagnosis, active case-finding, and patient centred care. These were identified as key learning area needs, and therefore included meeting course objectives and Outcome 1. Additionally, the training also included leading frameworks for TB elimination, such as the Search, Treat, and Prevent strategy [4,10,11,12,13]. Project development, including training in program theory, logic, and design, was a core component, supporting participants to develop and implement an “Award Project” (see “Participant Outputs”). Knowledge levels and individual learning objectives were assessed by surveys administered at the pre-course workshop. Findings were combined with AAI’s pre-specified learning objectives to inform the final design of the course content.
**Participant Outputs**	The main participant output was development and implementation of a project, designed by participants in response to perceived gaps in national-, provincial-, or district-level TB programs. By supporting participants’ learning skills in addition to knowledge growth, the projects were used as a learning tool to ensure that Course Outcome 2 was met. Participants initially developed a project proposal as part of their application to be accepted into the course. The topic was approved by the participant’s workplace supervisor. At the pre-course workshop, participants self-selected themselves into groups (18 across the three courses) on the basis of common interests or geographical location. They completed gap and solution analyses to revise concepts into a single group project. The final project topics were agreed at the end of the pre-course workshop with the delivery team’s support through problem and solution analyses. Daily group work during the in-Australia course focused on program theory and the identification and/or development of a project concept note, rationale, goal, objectives, activities to achieve objectives, indicators and means of verification, and a Gantt Chart and logical framework. The project implementation period was ~3 months between the course and the post-course workshop, at which participants presented a conference-style poster. This allowed for project evaluation and objective measurement of Course Outcome 2. After course completion, participants were eligible to apply for grants from AAI [14]. Some obtained funding support from workplaces or other avenues.
**Participant Selection**	Applicants from across Indonesia applied to AAI for acceptance into a course. An open scheme, whereby any eligible healthcare provider/policy-maker from Indonesia could apply online, and a nominated scheme for applicants from the Indonesian Ministry of Health, were both utilised. This approach was used to fulfil Objective 3—to ensure that course participants had a range of skills to help support the development of a multidisciplinary network, and provide cross-disciplinary analysis of program challenges in Indonesia. Applicants were short-listed, interviewed, then selected by a panel of health program specialists. The proposed project titles and interviews were used to assess the applicants’ technical knowledge and motivation. Selection criteria included support from the applicant’s place of work, development of a project concept, relevant qualifications, professional experience, the burden of TB in the applicant’s location, and whether participants represented areas nominated by the Ministry of Health as needing strengthening.
**Evaluation**	Course outcomes were evaluated by: surveys (1: attitudes to the course; 2: knowledge growth); the status of implementation of the project at the post-course workshop; and the networks forged and communication platforms established between participants, Indonesian experts, and Australian facilitators. The survey on attitudes to the course required participants to rank training relevance, satisfaction, and self-perceived project outcomes at the end of the course. Surveys to ascertain self-perceived knowledge growth were administered at the commencement of the pre-training workshop in Indonesia and the end of the course in Australia. Knowledge growth in 15 topics was assessed in a questionnaire ranking knowledge from zero (no knowledge) to 10 (complete knowledge). Qualitative surveys using in-depth interviews have been planned to be conducted by AAI approximately one year after completion of the last course to evaluate project implementation, determine longer-term course outcomes, understand lessons learned to help improve future courses, and inform future funding decisions regarding this style of training.

**Table 2:** Key training components and suggested recommendations for designing and delivering successful training.

Training Component	Recommendations
**Selecting training methodologies that enhance learning**	Teaching does not equate to learning. Adult participants learn best when the content is relevant to their job, their active participation is encouraged, motivations are supported, and experiences valued. Thus, training courses should incorporate participatory methods to encourage active learning, including [16]: Problem-based learning, such as problem analysis and solution design; Facilitated interactive discussions, such as debates or “meet the expert” question-and-answer sessions; Brief content presentations from a range of experts, reflecting the backgrounds of course participants (medical, nursing, microbiology, public health, academics, and non-government sector employees, such as advocacy experts); Group participatory sessions that emphasize “learning by doing”; Moderated group activities with presentations of work back to the group; Regular reflections of learning and methods of analysis; Site visits (if applicable); On-the-job learning, supervision, and support (if applicable).
**Fostering a positive learning environment**	Considerations for fostering a positive learning environment include: Allowing adequate time and space for all participants to have their opinions heard; Being aware that including senior healthcare staff may hinder discussions and questions, as some participants may feel that disagreeing with supervisors is disrespectful; Ensuring culturally appropriate and respectful training content and conduct from the training team throughout; If training is to be held in a country where participants do not live, having a welfare officer to coordinate and mediate participants and the training is advantageous.
**Selecting participants who have the ability and motivation to make a positive impact on TB programs**	Careful selection of participants can improve the likelihood that learning is translated into action within local TB programs (e.g., including healthcare providers and managers), and/or be sustainable with further knowledge translation opportunities (e.g., those with teaching or supervision roles). This can maximize the impact of training. A diverse and representative group of participants (e.g., across gender, ethnicity, health sector, age, and affected communities) should be included to enrich discussions.
**Ensuring participants understand and engage with training content**	Training needs to be provided in the participants’ first language (either directly or in simultaneous translation) unless English language fluency is high for all class members. Language skills need to be ascertained prior to course delivery. A pre-course workshop gives the course providers an opportunity to further gauge language competence of participants. This also ensures that bilingual language fluency is not an inhibitor for selection. In addition to translators, content experts who are fluent in the language of the participants are needed. Content experts can explain technical matters and pick up any misinterpretations. The content needs to be translated in advance by those who will translate in real time; dual projectors can then be used if slides also need to be shown in the language of course presenters. Beyond language, cultural competence and respect must be well-embedded in the design and delivery of the course.
**Creating content that is acceptable for varying baseline knowledge and areas of work**	Diversity in backgrounds and expertise of participants means that baseline knowledge and learning requirements may differ, and therefore it is difficult to pre-plan content. Asking participants to self-rank their knowledge on topics can help in pre-planning and adjusting the training content accordingly. Supporting students at different levels may include incorporating extra time for explaining concepts or providing relevant resources prior to the training for some, or providing thought-provoking, higher-level learning points to extend others. Group work that streams learners based on ability/knowledge could be considered. However, there are also advantages in mixing abilities/knowledge in group work activities to promote discussion and learning.
**Selecting dynamic and adaptable training methodologies**	Having multiple components to a training course is advantageous, such as the three-part structure of the AAI short course. It allows trainings to adapt to participant learning needs, and also gives an opportunity to measure retention of knowledge and impact. For example, a pre- and post-workshop can identify training needs before the training, and then evaluate training outputs, retention of knowledge, and behaviour changes after. Additionally, considering long-term, continued support for participants and, if possible, in-country supervision to promote positive training impacts is recommended [17].
**Evaluating and measuring training impact**	Training evaluations should be considered and organized prospectively. They should attempt to measure not just participant reactions, but also learning, behaviour change, and, although difficult, impact on TB programs or health systems [15].

## Supplementary Materials

The following are available online at https://www.mdpi.com/2414-6366/4/3/107/s1, Table S1: Participant Self-Evaluation of Learning Growth. Table S2: TB Elimination Post Training Survey. Table S3; Overview of projects developed by course participants.
