# Supplementary material for: Training for Tuberculosis Elimination in Indonesia: Achievements, Reflections, and Potential for Impact
Source: Trop Med Infect Dis. 2019 Jul 18;4(3):107. doi: 10.3390/tropicalmed4030107 (PMC6789479; doi:10.3390/tropicalmed4030107)
Supplement: Supplementary file 1 [file tropicalmed-04-00107-s001.zip › tropicalmed-04-00107-s001/Table S3- Overview of projects developed by course participants.docx]

| **Project Overview** | **Project Rationale** | **Implementation Area** | **Implementation Overview** |
| --- | --- | --- | --- |
| **Theme:** **Health Promotion and Adherence Support** | | |  |
| Peer support for TB patients and their families | TB patients and their families could play an important role in the community in TB prevention, but currently this potential is undervalued and underutilised. | Medan, Nagekeo, and Pulau Morotai | - Focus groups to investigate the needs of TB patients conducted - TB Peer Support Group established - Educational materials for facilitators and a TB education pocketbook for patients and families developed |
| ‘Puzzle TB’ – an adherence tool/game for TB patients to use to track adherence | A community health facility in Jayapura had high deafault rates. Patient knowledge of TB and TB treatment are low. | Mimika, Papua | - Puzzle in the shape of lungs created for drug-sensitive and drug-resistant TB regimens to illustrate planned treatment duration and provide a daily activity to cindicte with medication-taking. - Healthcare workers trained to use the Puzzle - Puzzle piloted in one community health centre |
| Creation of a comic book as a form of educational media for teenagers about pulmonary TB and its prevention | TB incidence among adolscents in Indonesia increased in 2017. Health promotion tools for adolscents in Indonesia, historically, have been poor. There was a need for relevant and engaging educational media for adolsccents. | Batang, Central Java | - Focus Group discussions held with adolscents to guide design, content and acceptability of the comic - Story and graphics designed - Book distributed to health services in Batung. |
| Increasing the treatment success rate of Papuan TB patients using educational films in the local languages (Amungme and Kamoro) | Many indigeneous Papuan people do not speak Indonesian fluently while the predminantly non Papuan healthcare workforce does not speak local languages. Past studies have shown that audio-visual health campaigns that use local languages were highly effective in raising awareness and assisting in treatment adherence. | Mimika, Papua | - Educational video using local Papuan actors filmed, focusing on the treatment journey and tackling stigma. - Video played in the local hospital waiting area and was intended to be distributed to various health centres and media platforms in Mimika |
| Development of counselling tools and a training package for staff, to use with patients living with TB | Papua Province has a high loss-to-follow-up rate. An improved level of understanding of TB along with adequate patient support would encourage treatment adherence. | Jayapura, Papua | - An information booklet was created for healthcare workers to assist in TB education and counselling - A training on how to use the booklet was conducted. - The booklet was piloted with TB patients |
| **Theme:** **Engaging the Private Sector** | | | |
| Implementation of a mandatory TB Case Notification system for Private GPs through training, incentives and a network with private and public facilities | A large number of TB cases are ‘missed’ each year in Indonesia as they are not reported by the private sector. There is a need to create, train and incentivise private facilities to notfiy TB cases to the National TB Program. | Surabaya, Java | - Simple mandatory notification system created - Provincial health staff and private sector staff, including provate GPs trained in the system - WhatsApp network created with private and public stakeholders, with quarterly meetings planned |
| Forming a TB partnership/network between primary care centres and private health facilities to increase the case notification rate, and build capacity and logistical support for private practices. | Formal collaboration is needed to ensure good recording and reporting of presumptive TB cases and patients in the private sector. | Rappocini, South Sulawesi | - Meetings between health authorities and private practitioners to discuss and agree on the project were held - Relevant notification and referral systems were developed |
| **Theme:** **Case finding initiatives** | | | |
| Use of village health cadres to increase index case contact investigation efforts and supporting a primary health facility to implement preventive therapy | Specific deficits in TB control in Indonesia are inadequate case finding efforts and implementation of preventive therapy. Most local community health centres in Medan were not meeting national targets. | Medan, North Sumatera | - Training and initiation of TB contact investigation by health cadres and health care workers - Persons with TB symptoms were reported and new TB cases were identified and started on treatment - There were many challenges with implementing preventive therapy in this period |
| Active case-finding by directly observed treatment, short-course (DOTS) supervisors at 2 primary health facilities | The implementation of active case finding by community health facilities is a major gap in the TB program in Indonesia. Largely due to adequate funding and human resources. | Malang, East Java | - TB contact investigation training for treatment supervisors and health care workers - Created an educational media to assist with contact investigation |
| Coordinating and training health professionals and nursing students to become active case finding officers | Jember community health facilities were not conducting active case finding due to limitations in human resources. | Jember, East Java | - Training of nursing students on how to perform contact investigation - Contact investigation and TB education/health literacy were performed by nursing students - 20 new TB cases were identified in the period |
| Development of a phone application to report TB case notifications to NTP | A large number of TB cases are not notified to authorities each year in Indonesia, a tool that could assist in finding persons with TB symptoms is required. | Kartasura, Central Java | - Design and development of an android application - The application underwent testing but needed additioanl funding to expand its development |
| To broaden coverage and improve quality of active case finding by cadres | In a district of Klaten, there were not adequate numbers of community health centre staff to implement and maintain active case finding. | Klaten, Central Java | - A mobile application for TB recording and reporting was created and piloted - Health cadres were trained as active case finding officers - Persons with TB symptoms were reported and new TB cases were identified |
| Use of village health cadres and primary health care nurses as active case finding teams and treatment supporters. | TB case finding in the project setting was not reaching national targets along with high loss to follow up rates. | Jayapura, Papua | - Cadres and nurses were trained and an active case finding team established - A simple ‘pocket’book with information about TB, the diagnostic/referral process and a treatment adherence monitoring sheet was created for the cadre/nurse-patient accompaniment system |
| Establishing and training village cadres, to undertake active case finding activities and contact investigation. | There was a lack of human resources and funding for a contact tracing team in the district. | Komodo, East Nusa Tenggara | - Village chief and stakeholders were engaged and an active case finding team created and trained - Household contact investigation was performed   Five new TB cases among contacts were diagnosed in the implementation period |
| **Theme:** **Preventive Therapy** | | | |
| Strengthening preventive therapy implementation among HIV (+) patients in primary care facilities | Coverage of TB preventive treatment among people living with HIV in Indonesia is very low. Current guidelines and policies are readily available in Indonesia, but they are not widely implemented in health centres. | Medan, North Sumatera | - Training of healthcare workers at primary care facilities in Medan on TB preventive therapy for people living with HIV - Providing relevant TB educational materials, recording and reporting forms and standard operating procedures |
| **Theme: Other** | | | |
| National TB guidelines phone application with diagnostic algorithm embedded | The National TB Guidelines are available online in pdf format for health practitioners to access. However many find it difficult to locate and/or navigate. | National | - An android mobile application was designed, developed and piloted nationally - Application launched and has been downloaded and used by hundreds of health practitioners across Indonesia |
| Improving the understanding of drug resistant (DR)-TB among pharmacists and expanding their role by monitoring DR-TB medicine prescriptions | Pharmacists play an important role in TB management, including DR-TB. Better monitoring of anti-TB prescription could prevent the development of DR-TB. | National | - An education module on DR-TB management for pharmacist was developed. - A training was held for hospital pharmacists in selected areas |
| Broadening the knowledge and improving the behaviour/attitude of hospital staff towards the process of recording and reporting of TB-HIV data via video tutorial | TB-HIV collaboration in hospitals has been a huge challenge. Under-reporting and under-recording, between TB and HIV units were identified as frequently reported issues. | Medan, North Sumatera & Tugurejo and Cilacap in Central Java | - Coordinated meetings between TB and HIV units to strengthen the collaboration and determine issues in recording and reporting - Held TB and HIV sessions to educate and inform hospital staff about TB and HIV - A focus group tested video tutorial was designed, developed, piloted and launched to teach healthcare workers how to correctly complete the TB/HIV recording and reporting forms. |
